# Rethinking feature reproducibility in radiomics: the elephant in the dark

**DOI:** 10.1186/s41747-025-00629-3

**Published:** 2025-09-04

**Authors:** Aydin Demircioğlu

**Affiliations:** Institute of Diagnostic and Interventional Radiology and Neuroradiology, University Hospital Essen, Essen, Germany

**Keywords:** Biomarkers, Machine learning, Radiomics, Reproducibility of results, Sample size

## Abstract

**Abstract:**

In radiomics, features are often linked to biomarkers and are generally expected to be reproducible, as reproducibility is considered a prerequisite for developing predictive models in clinical applications. However, this perspective overlooks feature interactions and may underestimate the potential value of nonreproducible features. Through experiments simulating a test–retest scenario, we demonstrate that even non-reproducible features can contribute significantly to predictive performance. Removing these features can lower model accuracy. These findings suggest that the emphasis on feature reproducibility should be reconsidered and that features should not be evaluated in isolation. Underlying information can be spread across multiple features. Focusing on individual features ignores feature interactions and may limit the model’s predictive power. Ultimately, radiomics must prioritize prediction and clinical relevance.

**Key Points:**

- Feature reproducibility assessments often ignore feature interactions, overlooking predictive performance.
- Feature reproducibility depends on subjective thresholds, chosen metrics, and sample size.
- Nonreproducible features can be more predictive than reproducible ones.
- Predictive information may be distributed across multiple features rather than confined to individual ones.

**Graphical Abstract:**

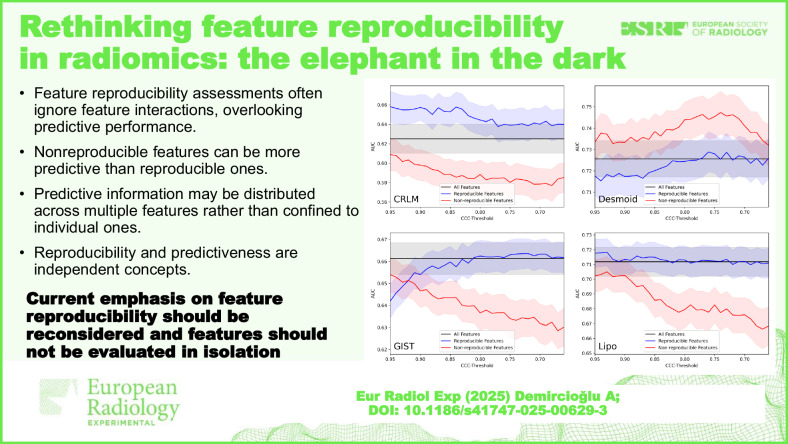

## Introduction

Rumi, the 13th-century Sufi poet, eloquently captured the limitations of individual perception in his well-known poem (translation by Coleman Barks [1], slightly modified):

*Some Hindus have an elephant to show*.

*No one here has ever seen an elephant*.

*They bring it at night to a dark room*.

*One by one, we go in*.

*One of us happens to touch the trunk. “A water-pipe kind of creature.”*

*Another, the ear. “A very strong, always moving back and forth, fan-animal.”*

*Another, the leg. “I find it still, like a column on a temple.”*

*Another touches the curved back. “A leathery throne.”*

*Another, the cleverest, feels the tusk. “A rounded sword made of porcelain.”*

*He’s proud of his description.*

*Each of us touches one place and understands the whole in that way.*

*The palm and the fingers feel in the dark are how the senses explore reality*.

*If each of us held a candle there, and if we went in together, we could see it.*

This parable, likely as old as humanity itself, illustrates a fundamental challenge in science: measurements are inherently noisy and often provide only a partial representation of reality.

We hypothesize that this is similarly true in radiomics, where quantitative features are extracted from medical images to act as proxies for underlying biological or pathological processes. Being considered biomarkers, these features are often evaluated only in isolation. This is particularly relevant for feature reproducibility studies, where the stability of individual features is assessed under varying conditions, such as different imaging parameters or segmentations from which the features are derived. In these studies, the stability of features is often framed as a prerequisite for clinical applicability, since a ‘nonreproducible’ feature could mean that during application, the data might vary in unexpected ways, leading to equally unexpected predictions. However, focusing solely on individual, reproducible features risks overlooking the critical role of feature interactions and could potentially lead to models that are unable to capture the underlying features and therefore perform worse.

We believe that considering individual features can only offer a fragmented view of the whole. The reality of radiomics, much like the elephant in the dark room, cannot be fully understood through isolated measurements.

## The elephant in the house

A simplified analogy further illustrates this point. Suppose we aim to determine whether an elephant is inside a house (here, the house could represent a patient, and the presence of an elephant could indicate the presence of a tumor). To make this determination, we examine multiple rooms, such as the living room, bedroom, and kitchen, each representing a feature. If the elephant is found in any room, meaning if any of the features is positive, we can confidently conclude its presence in the house.

**Fig. 1:**
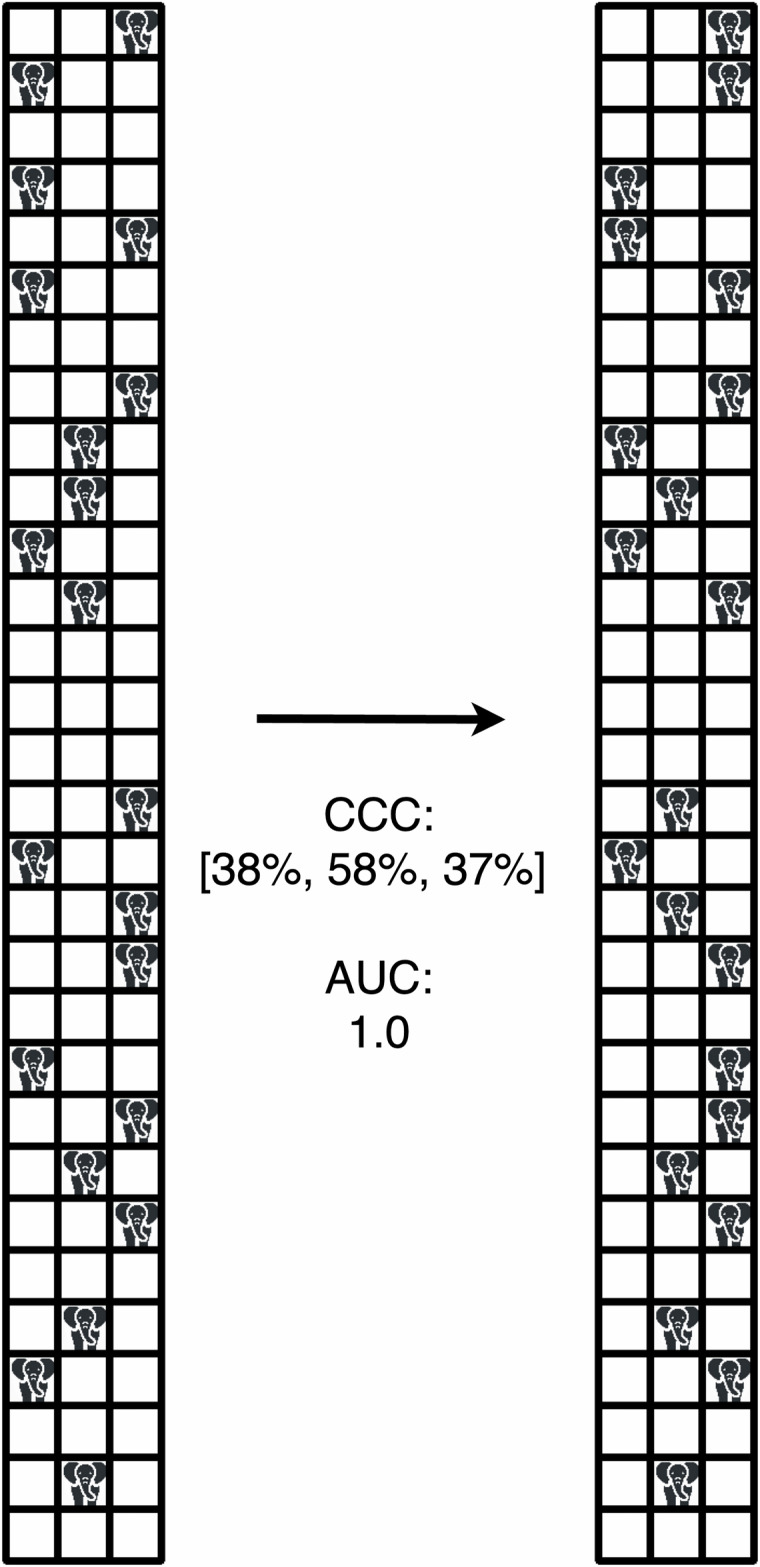
Illustration of the elephants in the house experiment. This schematic represents the “elephants in the house” experiment in a test–retest scenario. The grids show houses containing elephants at two timepoints (left and right), with each row representing a house and each cell representing a room. Since the elephants have moved between rooms in the second measurement (right), the three features corresponding to their locations would be considered non-reproducible based on common radiomics thresholds (> 0.70). However, the prediction remains highly accurate, as it relies on the presence of elephants. AUC, Area under the receiver operating curve; CCC, Concordance correlation coefficient

If we repeat the measurement, as studies in a test–retest scenario do, the elephants may, in the meantime, have moved between rooms (Fig. 1). This will cause individual features to vary strongly; however, it is *not* relevant *which* of the features is positive. As long as the elephant is inside the house, it will be in one of the rooms, and thus, one of the features will be positive, which is a highly accurate indicator of the presence of the elephant in the house. Yet, each feature, considered only by itself, will be nonreproducible. This simple example demonstrates that features may exhibit low reproducibility, yet still yield perfect predictive performance.

Building on this example, similar ones can be constructed. For instance, if tumor volume is predictive of malignancy but is assessed by measuring the area along slices of a computed tomography scan, the overall volume can still be reliably estimated as the sum of these individual measurements. However, in a test–retest scenario, slight shifts or rotations of the tumor could lead to large variations in the measured features. Despite their apparent lack of reproducibility, these features would still be highly predictive.

## Independence of reproducibility and predictiveness

Indeed, the concepts of reproducibility and predictiveness are independent of each other. Predictive features do not need to be reproducible if the underlying information is distributed across multiple features. Conversely, a reproducible feature does not need to be predictive. If a meaningless feature is used, for example, the shoe size of a patient, it can be highly reproducible without being at all predictive. The risk of having meaningless features is especially high in radiomics since many textural features (like Wavelet-HHH_GLCM_Contrast) are simply not humanly interpretable.

**Fig. 2:**
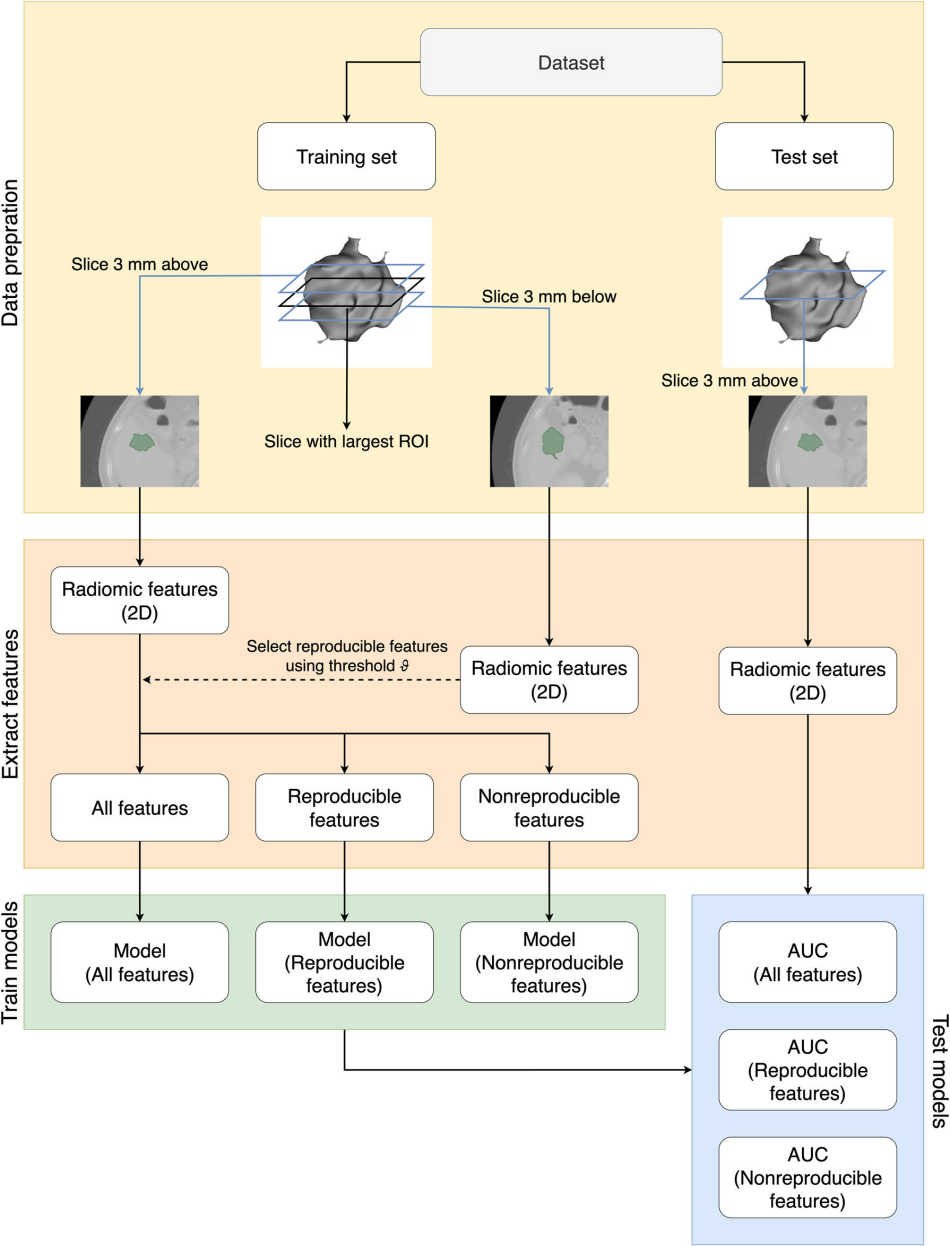
Study the flow of the reproducibility experiment. Each dataset was split into training and test sets. From the training data, slices 3 mm above the central slice were extracted. Reproducible features were identified by calculating the CCC with features from slices 3 mm below the central slice. Models were trained and tested on the hold-out test set using all, reproducible, and non-reproducible features. The threshold $\vartheta$ was varied between 0.60 and 0.95, and the experiment was repeated 100 times for each threshold. Modeling was performed using the least absolute shrinkage and selection operator (LASSO) and random forest. 2D, Two-dimensional; ROI, Region-of-interest

To further illustrate that nonreproducible features can still be predictive, we conducted an experiment using four datasets from the workflow for optimal radiomics classification (WORC) collection [2] (Table 1): Lipo, which contains MRI scans of patients with well-differentiated liposarcoma or lipoma; Desmoid, consisting of MRI scans from patients with desmoid-type fibromatosis or extremity soft-tissue sarcomas; CRLM, including CT scans of patients with liver metastases originating from colorectal cancer; and GIST, comprising CT scans of patients with gastrointestinal stromal tumors and similar intra-abdominal tumors. We closely mimicked a test–retest scenario (Fig. 2), in which a (sub)set of scans is repeated to identify reproducible features. However, for simplicity, instead of acquiring a second scan, we extracted slices and employed two-dimensional radiomics. Nearby slices can be considered as two measurements that are similar but not identical, akin to a test–retest scenario.

**Table 1:** Overview of the datasets

Dataset	Modality	Sample size	Size of minor class	Size of major class	Class-balance	In-plane resolution [mm]	Slice thickness [mm]
CRLM	CT	70	33	37	1.12	0.7 (0.6–0.9)	5.0 (1.0–8.0)
Desmoid	MR (T1)	190	68	122	1.79	0.7 (0.2–1.8)	5.0 (1.0–10.0)
GIST	CT	238	118	120	1.02	0.8 (0.6–1.0)	3.0 (0.6–6.0)
Lipo	MR (T1)	113	56	57	1.02	0.7 (0.2–1.4)	5.5 (1.0–9.1)

Each dataset was first preprocessed by resampling the images to an isotropic voxel size of 1 × 1 × 1 mm^3^ using B-spline interpolation. After stratified random splitting into training and testing folds, the provided segmentations, performed by expert radiologists, were used to identify the slices containing the largest region-of-interest (ROI), referred to as the central slice. We then created a dataset consisting of all slices located 3 mm above the central slice and used all slices located 3 mm below the central slice to measure the reproducibility of each feature using the concordance correlation coefficient (CCC) [3]. For this, 1015 radiomic features were extracted from each slice using PyRadiomics, with a bin width of 25, keeping all other extraction parameters at their default values. Image normalization using *z*-scores was applied for MRI datasets. Scans for which either the upper or lower slice did not contain a segmentation were excluded, as CCC could not be computed in these cases; this affected only a few scans. Using the CCC on the training set, we categorized the features into ‘reproducible’ and ‘nonreproducible’ sets based on a given threshold $\vartheta$.

We then trained predictive models using a combination of least absolute shrinkage and selection operator (LASSO) feature selection and a random forest classifier. These models were trained on the three different feature sets: all features, only reproducible features, and only nonreproducible features.

Model performance was subsequently evaluated by computing the area under the receiver operating characteristic curve (AUC) on the separated test set. To analyze the effect of feature reproducibility, we varied the threshold $\vartheta$ between 0.6 and 0.95. For each threshold value, we repeated the experiment 100 times with different train-test splits to assess variability in performance. In addition, we evaluated the area under the precision-recall curve (AUPRC), the F1 score, and sensitivity and specificity.

What can we expect from this simple experiment? If the reproducible features are indeed the most predictive, then we would expect that the model using these to outperform the model using all features. This is because nonreproducible features could be considered noise, increasing the overall complexity of the problem, making modeling more difficult, and ultimately leading to lower predictive performance. Additionally, the model trained only on nonreproducible features should perform worse than both other models.

**Fig. 3:**
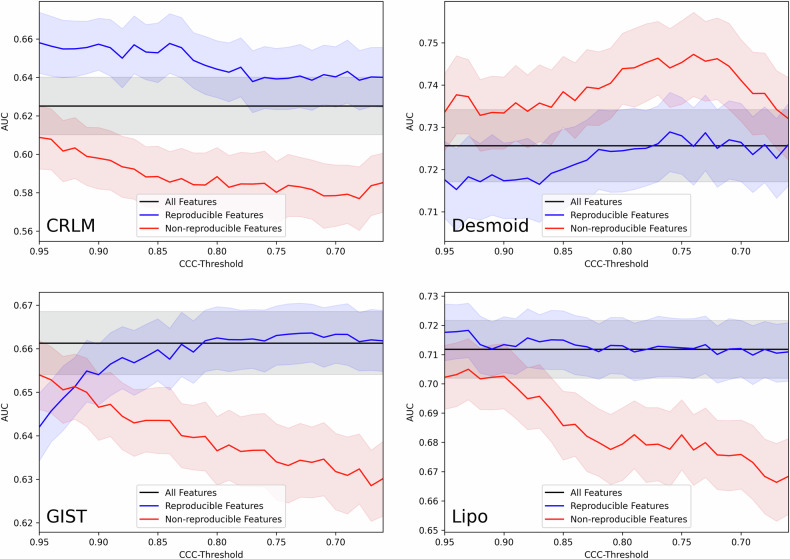
Results of the reproducibility experiment, evaluated by AUC. In each plot, the AUCs of the models using all features (black), reproducible features (blue), and nonreproducible features (red) are shown for each threshold. The experiment was repeated 100 times, and the 95% confidence interval of the average AUC is shown as a shaded region. Only the CRLM dataset’s results align with expectations, where models using only reproducible features improve generalizability. For the Desmoid dataset, nonreproducible features outperform reproducible ones. In the GIST and Lipo datasets, models using all features perform as well as those using only reproducible features. AUC, Area under the receiver operating characteristic curve; CCC, Concordance correlation coefficient

Indeed, this is what we observe for AUC, but only on one of the four datasets, CRLM (Fig. 3). In contrast, the Desmoid dataset exhibits the opposite trend: here, the nonreproducible features outperformed the reproducible ones, particularly when using a threshold around 0.75. This suggests that removing the most reproducible features results in a better-performing model. A similar pattern was observed in the GIST dataset, though only for threshold values up to around 0.92. Moreover, a closer examination of plots (except CRLM) revealed that the model using the most reproducible features does not outperform the model using all features, contradicting our initial expectations. Finally, in the Lipo dataset, the nonreproducible features behave as expected across all thresholds, aligning with our expectations.

The results for AUPRC and F1 closely mirror those observed with AUC, while sensitivity and specificity are less consistent, likely because these metrics rely on the optimal point of the ROC curve and tend to be less robust (Supplementary Materials, Figs. S1–S4).

## Discussion

This experiment vividly illustrates that distinguishing between ‘reproducible’ and ‘nonreproducible’ features is not straightforward. Since measurements are inherently noisy, no feature can be fully reproducible, which necessitates the application of a subjective threshold [4]. The choice of this threshold is further influenced by its dependence on the selected metric, such as the intraclass correlation coefficient or CCC [5]. Despite its importance, little research has explored how these choices influence which features are considered reproducible.

**Fig. 4:**
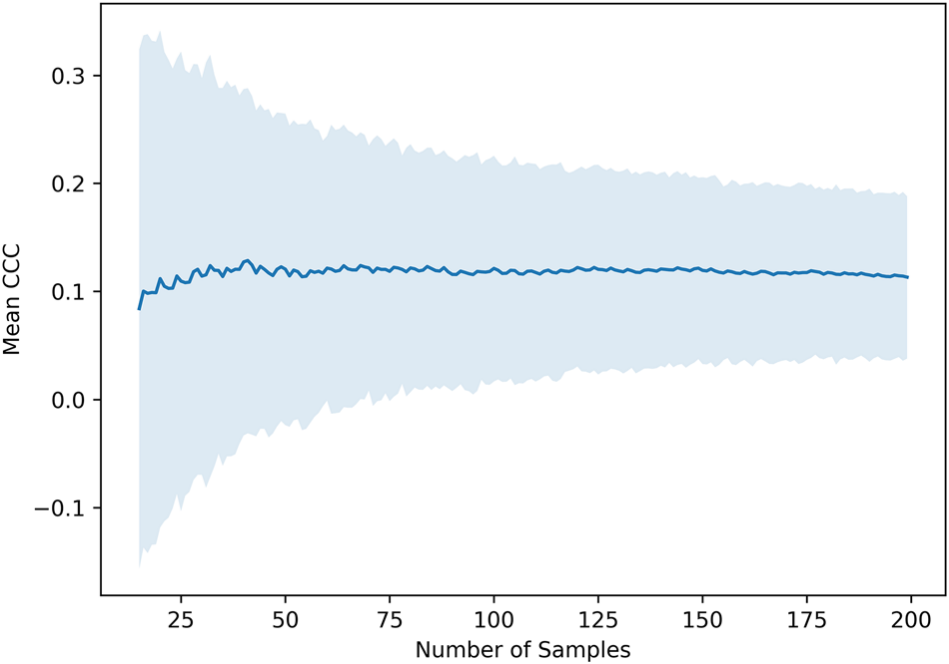
Dependence of the mean CCCs on the sample size. The sample size in the elephant experiment using three features was deliberately varied between 15 and 200. The experiment was repeated 100 times, and the 95% confidence interval of the average observed CCC is shown as a shaded region. Significant variation is observed, particularly for smaller sample sizes, raising concerns about the reliability of test–retest scenarios in such cases. CCC, Concordance correlation coefficient

Low sample sizes, a common issue in radiomics, exacerbate this uncertainty [6]. Indeed, when we vary the sample size in the ‘elephant in the house’ experiment with three features and 100 repeats, a high level of uncertainty in the average CCC is revealed (Fig. 4). This suggests that reproducibility studies based on small cohorts with fewer than 50 samples may lead to unreliable conclusions about which features are genuinely reproducible [3].

Moreover, given the independence of the concepts, classifying features as ‘reproducible’ does not necessarily imply greater predictive power compared to those classified as ‘nonreproducible’. The relevant information may be distributed across multiple features, and limiting analysis to those deemed reproducible could lead to suboptimal models.

These findings contrast with the prevailing view that feature reproducibility is a prerequisite “[…] for model development and subsequent analyses” [7]. In one of the first studies on reproducibility of radiomics features, it was stated that they “must be reproducible to qualify as biomarkers for clinical care” and that “image features with higher reproducibility will have the potential to distinguish smaller differences” [4]. As a result, many studies on feature reproducibility overlooked the assessment of predictiveness. Even if reproducible features were perfectly identified, modeling typically relies on machine learning. Many commonly used models, such as support vector machines or random forests, are considered ‘black boxes’ and cannot be readily interpreted by humans, necessitating explanations. Intriguingly, these explanations are only meaningful if the model performs well, as explanations for a nonpredictive model are uncertain. This suggests that a highly predictive model is a prerequisite for analyzing feature reproducibility. This issue is further amplified by the abstract nature of many radiomic features, which lack clear interpretation.

We believe that, at present, a univariable perspective, in which the interaction between features is overlooked, is prevalent in radiomics. This is reflected in the concept of a ‘biomarker’, which, by definition, ‘must be informative, or sensitive to underlying biology, as well as reproducible and reliable across various image acquisition settings and quantitative methods’ [8]. However, as demonstrated in our examples, this idealistic view is rarely reflected in real data. Radiomic features are typically highly correlated due to the application of preprocessing filters [9], making biomarker identification without experimental validation questionable. The field remains largely exploratory in nature, and empirical studies are still largely lacking [10].

Feature reproducibility must also be considered in the context of the entire radiomics pipeline. Identifying a single best-performing model is often infeasible due to the Rashomon effect, wherein multiple models achieve similar performance [11, 12]. However, these models often utilize vastly different features, making it principally unclear which should be regarded as ‘correct’. Even seemingly minor differences in normalization will result in largely different feature importances for the resulting model [13, 14]. It is important to remember the saying that while all models are wrong, a few are still useful [15].

It is interesting to compare radiomics to deep learning. Historically, features played a crucial role in image processing, with concepts such as texture and wavelet features, currently used in radiomics, originating from this field. However, these approaches are not widely used today, as they have been shown to be inferior in terms of performance compared to deep learning, where feature generation is data-specific. They are also not employed for interpreting neural networks. Instead, current approaches focus on the images themselves, aiming to understand which regions influenced the network’s predictions. A similar shift, involving habitat radiomics [16] and feature maps [17], warrants thorough exploration in radiomics.

Our analysis was conducted using internal test data, which, due to the resampling method, followed the same distribution as the training data. In general, if new data does not follow the same distribution, it is unclear how the model will perform since this will depend on the specific case. Therefore, it remains unclear whether our results hold for external data. However, a recent study by Wennmann et al [18] compared models to predict plasma cell infiltration in patients with monoclonal plasma cell disorders that used either all features or only reproducible features, among others, in terms of their performance on external datasets. They demonstrated that while the model using reproducible features showed the best generalization, it was outperformed by the model using all features. Contrary to their expectations, this could indicate that the less stable textural features contributed markedly to the performance of the model.

While these results align with our hypothesis, neither their experiments nor ours explain the different patterns observed across the four datasets considered here. It is reasonable to expect that these patterns might depend on the type of tumor, however, for any deeper insight, a similar study must be performed on a much larger collection of radiomic datasets. Unfortunately, such large data collection does not currently exist in radiomics, which remains a major challenge for the field and results in significant variations in the observations [19]. Until such analysis is performed, no clear recommendations can be given regarding whether to exclude nonreproducible features.

Clearly, our observations do not invalidate studies on feature stability and reproducibility. While our analysis focused on test–retest reliability, we believe that similar considerations may apply to other assessments of feature reproducibility, such as segmentation variability. Further investigations across diverse radiomic datasets are necessary.

Our findings challenge the idea that feature reproducibility is a prerequisite for predictive models. They also demonstrate that underlying information can be spread across multiple features. Focusing on individual features ignores feature interactions and may limit the model’s predictive power. Ultimately, radiomics must prioritize prediction and clinical relevance.

## Supplementary information

**Additional file 1**: **Fig. S1.** Results of the reproducibility experiment, evaluated by AUPRC. In each plot, the AUPRCs of the models using all features (black), reproducible features (blue), and nonreproducible features (red) are shown for each threshold. The experiment was repeated 100 times, and the 95% confidence interval of the average AUPRC is shown as a shaded region. AUPRC, Area under the precision-recall curve; CCC, Concordance correlation coefficient. **Fig. S2.** Results of the reproducibility experiment, evaluated by F1 score. In each plot, the F1 scores of the models using all features (black), reproducible features (blue), and nonreproducible features (red) are shown for each threshold. The experiment was repeated 100 times, and the 95% confidence interval of the average F1 score is shown as a shaded region. CCC, Concordance correlation coefficient. **Fig. S3.** Results of the reproducibility experiment, evaluated by sensitivity. In each plot, the sensitivity of the models using all features (black), reproducible features (blue), and nonreproducible features (red) are shown for each threshold. The experiment was repeated 100 times, and the 95% confidence interval of the average sensitivity is shown as a shaded region. CCC, Concordance correlation coefficient. **Fig. S4.** Results of the reproducibility experiment, evaluated by specificity. In each plot, the specificity of the models using all features (black), reproducible features (blue), and nonreproducible features (red) are shown for each threshold. The experiment was repeated 100 times, and the 95% confidence interval of the average specificity is shown as a shaded region. CCC, Concordance correlation coefficient.

## Data Availability

The datasets generated and/or analyzed during the current study are available in the GitHub repository at https://github.com/aydindemircioglu/radRepro.

## Abbreviations

AUC: Area under the receiver operating curve
CCC: Concordance correlation coefficient
