# Supplementary material for: Rethinking feature reproducibility in radiomics: the elephant in the dark
Source: Eur Radiol Exp. 2025 Sep 4;9:85. doi: 10.1186/s41747-025-00629-3 (PMC12411371; doi:10.1186/s41747-025-00629-3)
Supplement: Supplementary file 1 — Additional file 1: Fig. S1. Results of the reproducibility experiment, evaluated by AUPRC. In each plot, the AUPRCs of the models using all features (black), reproducible features (blue), and nonreproducible features (red) are shown for each threshold. The experiment was repeated 100 times, and the 95% confidence interval of the average AUPRC is shown as a shaded region. AUPRC, Area under the precision-recall curve; CCC, Concordance correlation coefficient. Fig. S2. Results of the reproducibility experiment, evaluated by F1 score. In each plot, the F1 scores of the models using all features (black), reproducible features (blue), and nonreproducible features (red) are shown for each threshold. The experiment was repeated 100 times, and the 95% confidence interval of the average F1 score is shown as a shaded region. CCC, Concordance correlation coefficient. Fig. S3. Results of the reproducibility experiment, evaluated by sensitivity. In each plot, the sensitivity of the models using all features (black), reproducible features (blue), and nonreproducible features (red) are shown for each threshold. The experiment was repeated 100 times, and the 95% confidence interval of the average sensitivity is shown as a shaded region. CCC, Concordance correlation coefficient. Fig. S4. Results of the reproducibility experiment, evaluated by specificity. In each plot, the specificity of the models using all features (black), reproducible features (blue), and nonreproducible features (red) are shown for each threshold. The experiment was repeated 100 times, and the 95% confidence interval of the average specificity is shown as a shaded region. CCC, Concordance correlation coefficient. [file 41747_2025_629_MOESM1_ESM.pdf]

Rethinking feature reproducibility in radiomics: the elephant in the dark

ELECTRONIC SUPPLEMENTARY MATERIAL

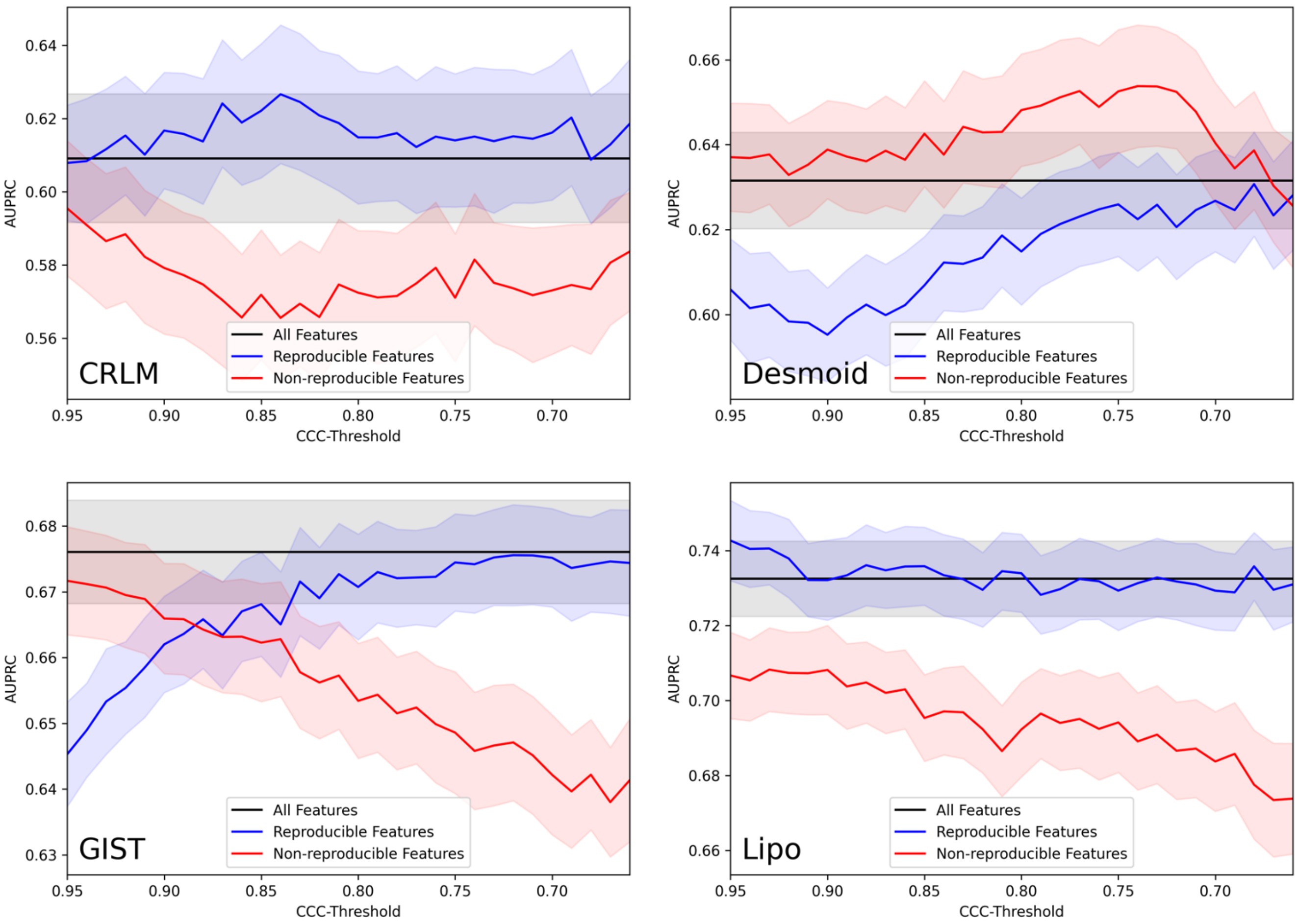

Fig. S1 Results of the reproducibility experiment, evaluated by AUPRC. In each plot, the AUPRCs of the models using all features (black), reproducible features (blue), and non-reproducible features (red) are shown for each threshold. The experiment was repeated 100 times, and the 95% confidence interval of the average AUPRC is shown as a shaded region. *AUPRC* Area under the precision-recall curve, *CCC* Concordance correlation coefficient.

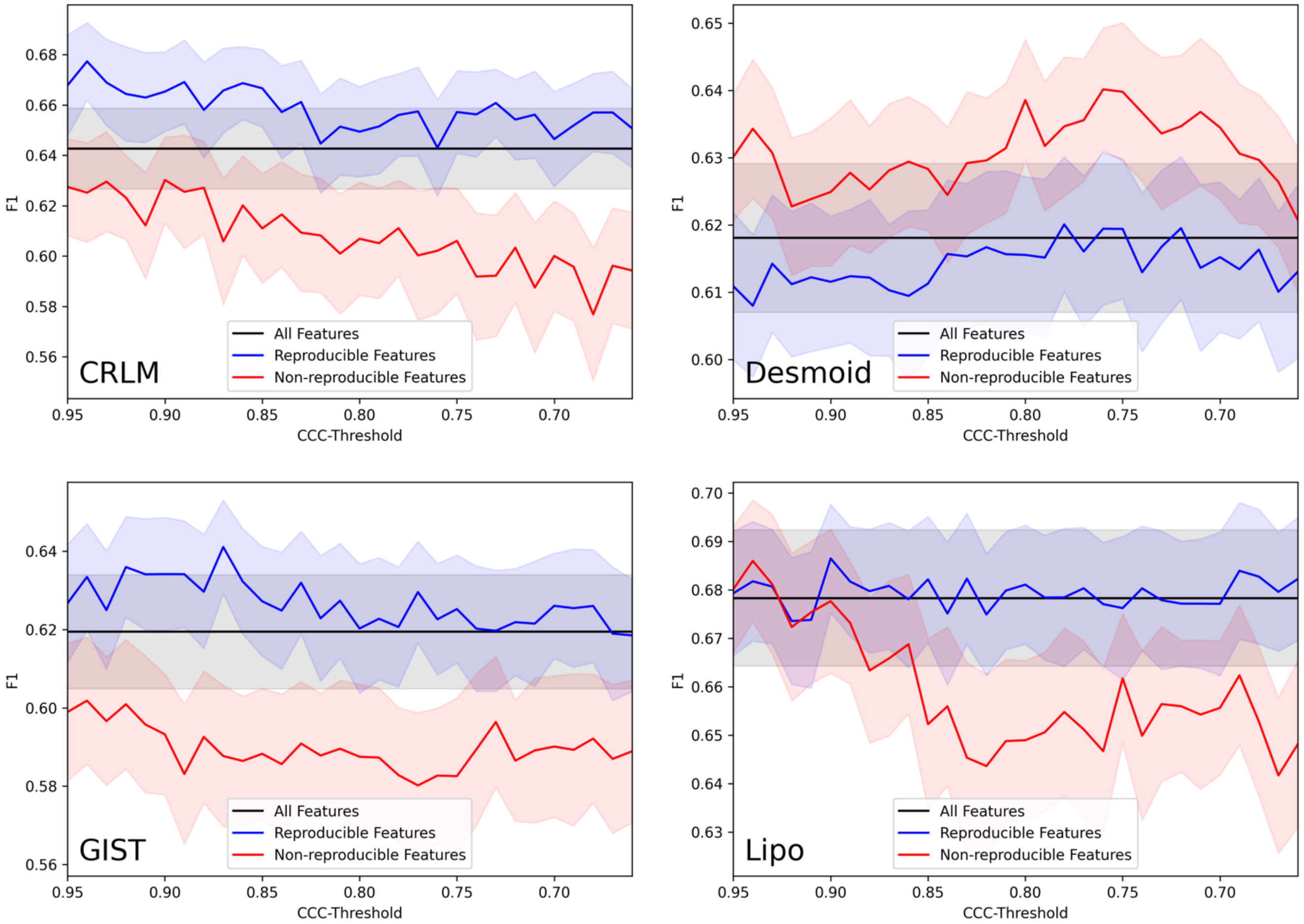

Fig. S2 Results of the reproducibility experiment, evaluated by F1 score. In each plot, the F1 scores of the models using all features (black), reproducible features (blue), and non-reproducible features (red) are shown for each threshold. The experiment was repeated 100 times, and the 95% confidence interval of the average F1 score is shown as a shaded region. *CCC* Concordance correlation coefficient.

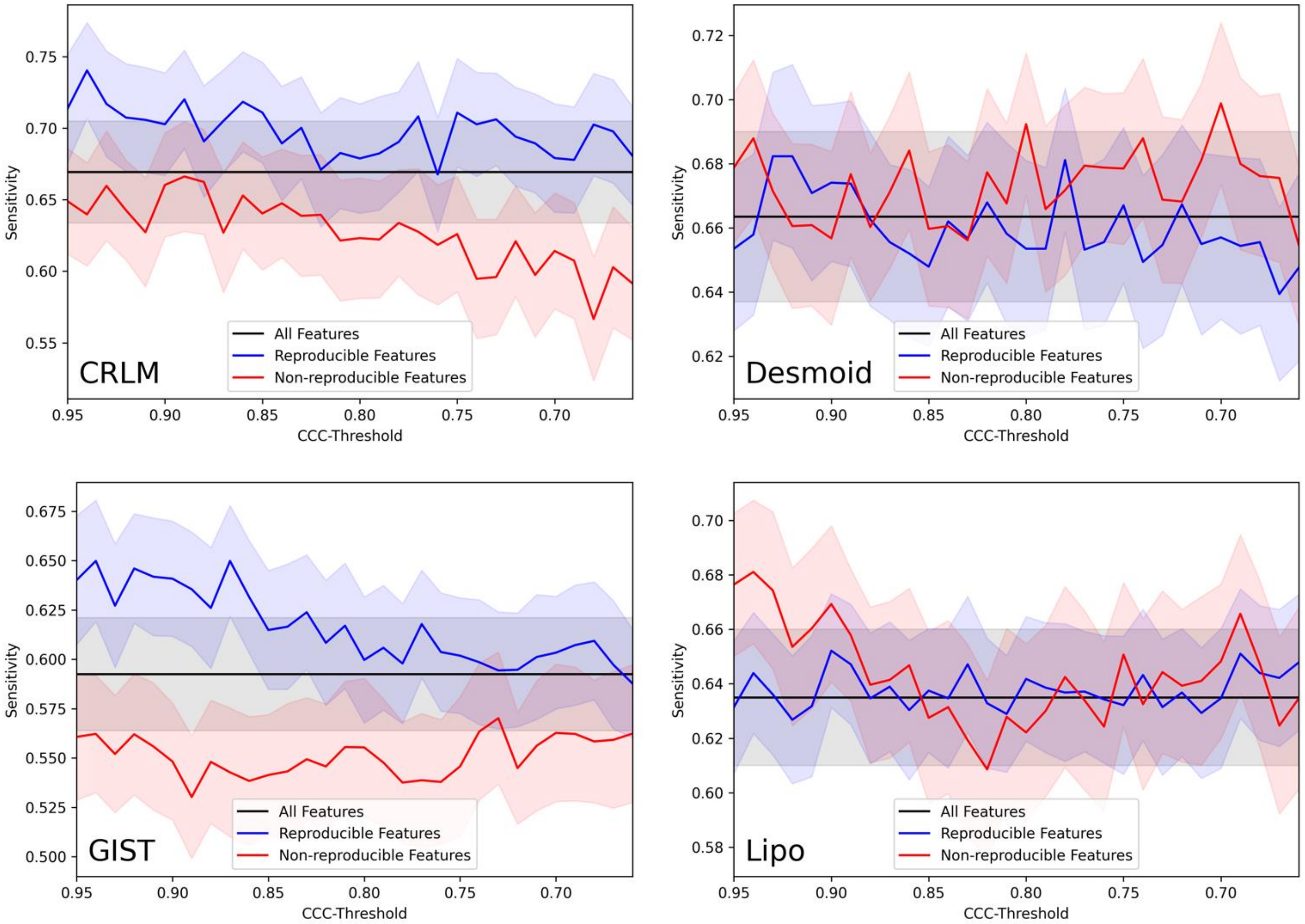

Fig. S3 Results of the reproducibility experiment, evaluated by sensitivity. In each plot, the sensitivity of the models using all features (black), reproducible features (blue), and non-reproducible features (red) are shown for each threshold. The experiment was repeated 100 times, and the 95% confidence interval of the average sensitivity is shown as a shaded region. *CCC* Concordance correlation coefficient.

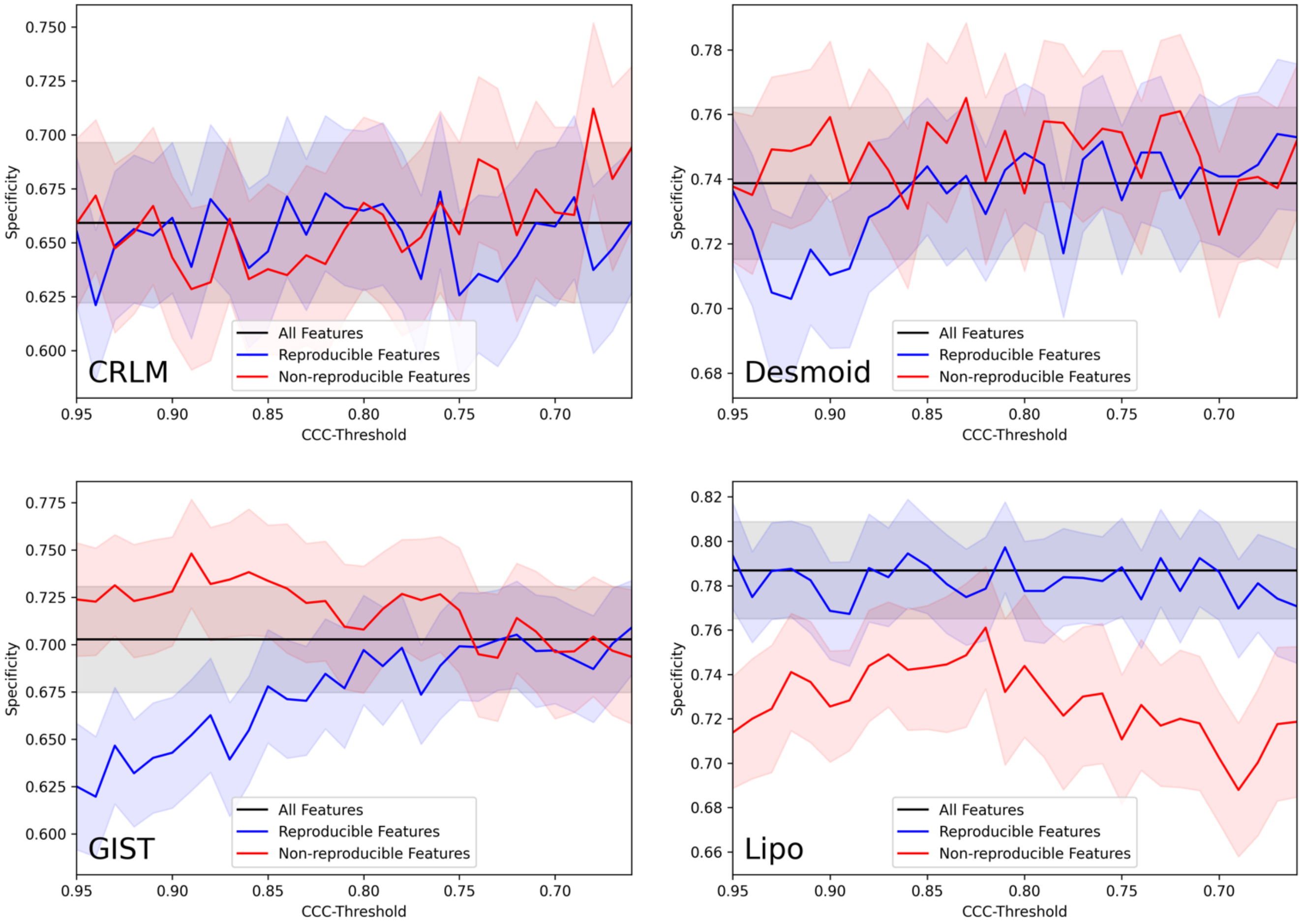

Fig. S4 Results of the reproducibility experiment, evaluated by specificity. In each plot, the specificity of the models using all features (black), reproducible features (blue), and non-reproducible features (red) are shown for each threshold. The experiment was repeated 100 times, and the 95% confidence interval of the average specificity is shown as a shaded region. *CCC* Concordance correlation coefficient.
